# Methods2AOP: A Collaboration to Strengthen the Integration of Test Methods into the Adverse Outcome Pathway Framework

**DOI:** 10.12688/f1000research.172881.1

**Published:** 2025-12-08

**Authors:** Agnes L. Karmaus, William Bisson, Albert Braeuning, Xiaoqing Chang, Laure-Alix Clerbaux, Julija Filipovska, Jennifer Fostel, Ksenia Groh, Ginnie Hench, Eftychia Lekka, Scott G. Lynn, Kelly A. Magurany, Kristan Markey, Anna Maria Masci, Holly Mortensen, Jason M. O'Brien, Emily Reinke, Nyssa Tucker, Vassilis Virvilis, Barbara Viviani, Sara Vliet, Clemens Wittwehr, Helena T. Hogberg

**Affiliations:** 1 Inotiv, Research Triangle Park, USA; 2 Bundesinstitut fur Risikobewertung, Berlin, Berlin, Germany; 3 Universite catholique de Louvain Institut de Recherche Experimentale et Clinique, Louvain, Belgium; 4 Independent Researcher, Ohrid, North Macedonia; 5 National Institute of Environmental Health Sciences, Research Triangle Park, North Carolina, USA; 6 Eawag Swiss Federal Institute of Aquatic Science and Technology, Zurich, Switzerland; 7 OpenBioDataModeling, Research Triangle Park, USA; 8 Biovista, Athens, Greece; 9 US Environmental Protection Agency, Washington, USA; 10 Verto Solutions LLC, Washington DC, USA; 11 The University of Texas MD Anderson Cancer Center, Houston, Texas, USA; 12 Environment and Climate Change Canada National Wildlife Research Centre, Ottawa, Ontario, Canada; 13 The University of North Carolina at Chapel Hill, Chapel Hill, North Carolina, USA; 14 Universita degli Studi di Milano-Bicocca, Milan, Lombardy, Italy; 15 European Commission Joint Research Centre, Ispra, Italy

**Keywords:** Adverse Outcome Pathways (AOPs), New Approach Methods (NAMs), regulatory framework, ontologies

## Abstract

The Adverse Outcome Pathway (AOP) framework is a pivotal tool for organizing mechanistic knowledge and linking it to adverse outcomes of regulatory significance. However, the integration of test method information, particularly New Approach Methods (NAMs), within the central repository for AOP knowledge, (the AOP-Wiki), has been suboptimal, limiting the framework’s utility for regulatory decision-making. The Methods2AOP collaboration, comprised of various international stakeholders, was established to address this gap and enhance the role of test methods within the AOP framework. This paper reviews their work emphasizing the importance of linking detailed test method information and conceptually proposes how it may be included in the AOP knowledgebase. The Methods2AOP collaboration proposes using ontologies to standardize and structure information, thereby facilitating interoperability, enabling reusability, and establishing clear connections between test methods and Key Events (KEs). A conceptual model is presented to demonstrate qualitative similarities between concepts in key event components and structured methods information. The implementation of Methods2AOP recommendations would increase the clarity and transparency of method descriptions, which could support regulatory acceptance and a wider adoption of NAMs. The broad community of stakeholders impacted by this work stands to benefit from the Methods2AOP recommendations through enhanced regulatory decisions, increased visibility and scientific impact, new market opportunities, and the accelerated adoption of NAMs in regulatory affairs. In summary, the Methods2AOP collaboration presents a comprehensive effort to formally standardize the integration of test methods into the AOP framework, thereby fostering a more robust, and transparent system that aligns with the goals of the scientific and regulatory communities.

## Plain language summary

New approach methods (NAMs) are state-of-the-science methods that can provide mechanistic insight into chemical safety instead of relying on traditional whole animal test systems alone. The adverse outcome pathway (AOP) is a framework that combines data from various sources including NAMs and traditional studies, and can therefore link mechanisms to adverse outcomes. This helps building confidence in using NAMs instead of animals in hazard and risk assessment. AOPs can be found in the AOP-Wiki, a web interface that includes information supporting the AOP. However, it contains limited information about the actual test methods. The Methods2AOP collaboration was initiated to identify, prioritize, organize and standardize methods information using a harmonized language. The implementation of the Methods2AOP collaboration is expected to provide a powerful and transparent system that can support the use of NAMs and the AOP framework in various contexts, including hazard and risk assessment.

## 1. The adverse outcome pathway framework: Integrating data sources for hazard and risk assessment

### 1.1 Background

The Adverse Outcome Pathway (AOP) conceptual framework was developed to address the need of the regulatory and scientific community to better organize and streamline existing toxicological mechanistic knowledge, and link to adverse outcomes (AO) of regulatory significance ( Ankley et al., 2010). Moreover, AOPs outline a specific cascade and assess the evidence related to the essentiality of particular biological perturbations (i.e., molecular initiating events (MIEs) and key events (KEs)) and causality of the linkages between them (key event relationships (KERs)), thus proposing a plausible pathway leading to an AO at the organ, organism and/or population level. The AOP framework focuses on the evidence for linkages between the critical/essential KEs in a stressor-agnostic approach ( Villeneuve et al., 2014; Clerbaux et al., 2024). This supports integration of various data sources (Integrated Approaches to Testing and Assessment - IATA) including new approach methods (NAMs) that can detect and measure a KE at lower organizational levels, i.e., at the molecular and cellular level leading to an AO at the organizational level of interest. A benefit in developing and applying AOPs is to build confidence in using NAMs data to characterize trajectory to adversity, specifically hazard for an endpoint, without using whole animals. Once the AOP is well-established, several NAMs that measure the various KEs can be combined into a defined approach (DA) or IATA to provide an actionable framework for the use of AOPs. The newly adopted OECD Guideline No. 497: Defined approaches on Skin Sensitization is an example where the AOP framework has contributed to the acceptance of NAMs in a regulatory application ( OECD, 2023b). However, more complex endpoints, e.g., carcinogenicity and developmental neurotoxicity (DNT), require networks of many AOPs that still need to be enhanced ( Pitzer et al., 2023; OECD, 2023a; Sanz-Serrano et al., 2024). Application of NAMs for hazard evaluation does not require AOPs. However, well-developed and endorsed AOPs can help increase confidence in NAMs and support regulatory use by the organisation of test batteries that address events critical to the progression to a regulatory relevant AO ( ICCVAM, 2024; Bajard et al., 2023; van der Zalm et al., 2022).

The scientific development of NAMs and their uptake for mechanistic investigation are steadily progressing ( Bisson et al., 2024), yet application in toxicological risk assessment is lagging. To align the progress of method development and mechanistic assay use with the acceptance for regulatory application, more interaction between the method developers, regulatory communities and end users is needed ( van der Zalm et al., 2022; ICCVAM, 2018). To support this communication and bolster the role of AOPs in regulatory application, the international Methods2AOP collaboration was established to develop a formal proposal for the integration of structured methods documentation for AOPs. Given the fundamental premise that the KEs in an AOP must be measurable, methods have a crucial role and therefore should be robustly captured in the AOP description, which in turn can help with transparency and bolster regulatory confidence ( Carusi et al., 2022).

### 1.2 Current AOP infrastructure

Paramount to the undertaking of the Methods2AOP effort was a clear understanding of the current AOP infrastructure and uses. With a future goal of integrating additional information into the schema, a diverse group of stakeholders were engaged to leverage past experiences. It is important to distinguish the Methods2AOP collaboration’s method-centric scoping within the AOP infrastructure from the existing AOP-knowledgebase (AOP-KB) as the intention was to identify information that could add to and refine the existing infrastructure, not to re-build established interfaces.

**Figure 1.**
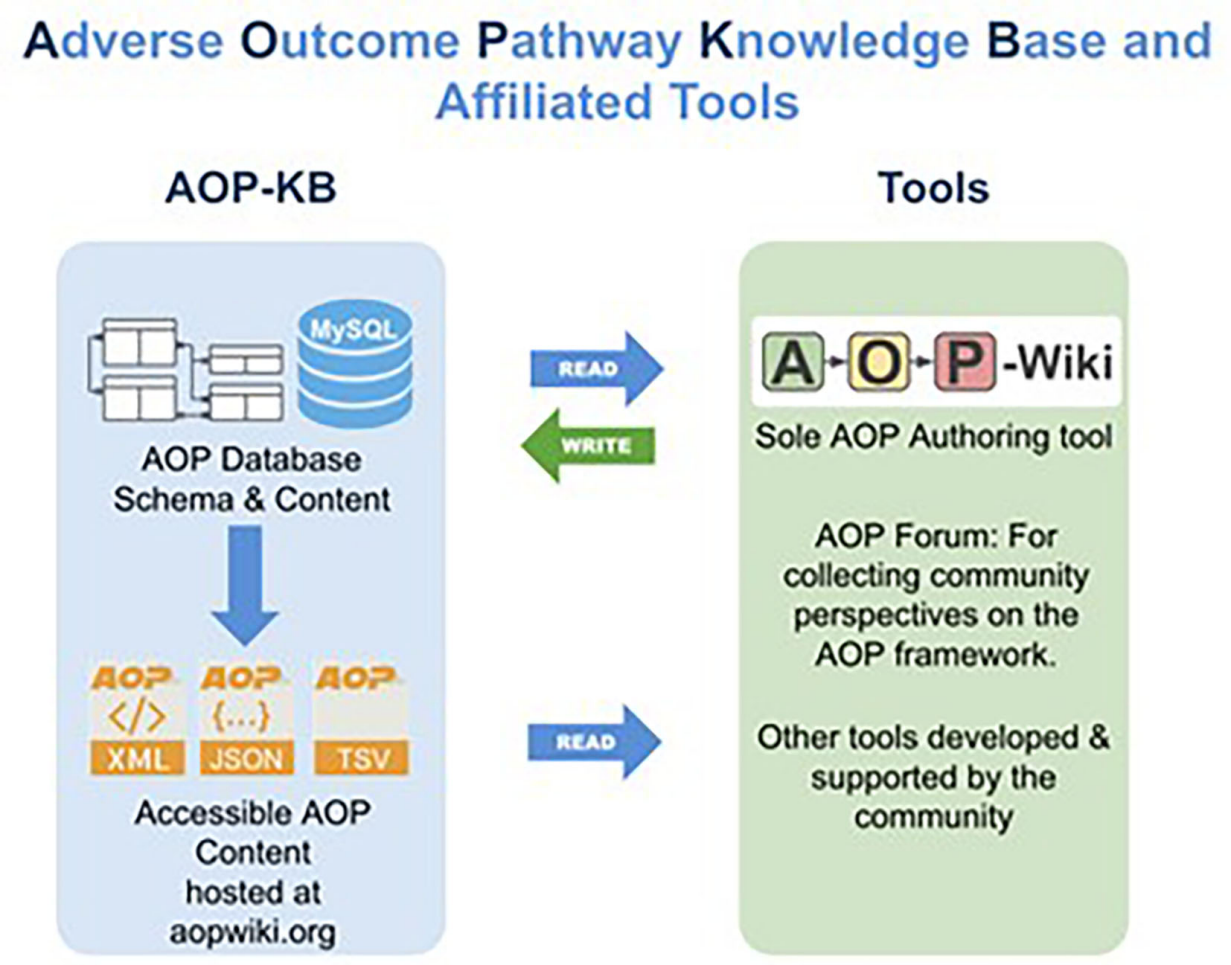
Database connection of the AOP Knowledge Base, AOP Knowledge Base Tools, and the AOP-Wiki. The AOP-KB (left side) is the primary central repository for all AOPs developed either as part of the OECD AOP Programme, or by the larger scientific community. The AOP-KB Tools (right side) are a collection of web-based resources that are constantly undergoing development and refinement.

The central location used for managing, and disseminating AOP knowledge is the AOP-KB ( Figure 1), ^1^ the database underlying the AOP-Wiki. ^2^ The AOP-KB and AOP-Wiki are administerd by the AOP-KB Coordination Group under the auspices of the AOP Program ^3^ of the Organisation for Economic Co-operation and Development (OECD). The AOP-Wiki, the official resource for creating and editing AOP content, has been available since 2012 and is currently on version 2.7 with roughly 1000 registered users working on more than 500 AOPs. Many more non-registered users access the AOP-Wiki in read-only mode to view its content. The AOP-KB content, exposed in XML, JSON, and TSV formats through the AOP-Wiki, serves as a resource used by third party tools, a collection of web-based resources, developed by AOP community partners that are constantly undergoing development and refinement.

The Methods2AOP collaboration suggest content and a conceptual documentation schema, presented herein for future consideration to integrate into the AOP-KB. It is important to consider that any future changes made to the AOP-KB and interface of the AOP-Wiki would require considerable effort and restructuring that will ultimately have an impact on a variety of third-party tools. There are other ongoing efforts aimed at improving AOP standards within the AOP-KB ( Hench et al., 2024b), such as the Environmental Health Language Collaborative (EHLC) AOP Standards Workshop, ^4^ the FAIR AOP Cluster working group ( Mortensen et al., 2025) and the ELIXIR Toxicology Community, ^5^ that all need to be aligned. Ultimately, if revisions to the AOP-KB structure and data model relationships are made to reflect integration of methods information, it will need to be conducted thoughtfully with all tools that draw from the centralized AOP-KB in mind. This way updates can be pushed to ensure all official tools and even third-party tools can keep up to date.

In the current AOP-Wiki interface (representing database fields accounted for in AOP Data and mapped in the Data Model), AOP authors can provide qualitative information on KEs and appraise the evidence and quantitative understanding (high, moderate, low) of their linkages (i.e., KER) according to the Guidance Document on Developing and Assessing AOPs, ^6^ and its practical supplement, the Developers’ Handbook. ^7^ However, test methods are currently not documented in the AOP-Wiki in a systematic way, rather, AOP authors can fill in an unstructured free text section “How It Is Measured or Detected” (“It” being the relevant KE) to provide any information they chose regarding test method(s). According to the Developers’ Handbook, ^6^ this section is not intended to provide detailed protocols but to capture the type of output that can be employed to evaluate the KE and the level of scientific confidence in the underlying measurements. This does not reflect the important role of the test methods, and does not allow compiling and sharing structured information. The lack of structured documentation for test methods limits transparency and creates inconsistency, which potentially makes it difficult for users (e.g., regulators) to understand the relevance and the reliability of the KE.

## 2 Role Served by the Methods2AOP collaboration

### 2.1 Establishing the Methods2AOP collaboration

One of the fundamental principles within the AOP community is “crowdsourcing”: the voluntary and result-driven temporary collaboration of a group of individuals to reach a common goal. Crowdsourcing means tapping into a big, diverse group of people to gather their ideas, skills, or knowledge for a specific purpose. Instead of relying on just a few experts, numerous different people can be invited to contribute their thoughts or talents. This is institutionalized in the AOP framework which aims at getting a wide range of input (recorded in MIEs, KEs, AOs and KERs) that might not be available in any other fashion. By its nature, crowdsourcing needs participants who are willing to pool their expertise and actively collaborate with others to produce relevant AOPs ( Carusi et al., 2023). When the goal of addressing test method descriptions in the AOP-Wiki was identified, it was therefore natural that the same crowdsourcing concept be applied to building the Methods2AOP collaboration. Participants from interested organizations were assembled to formulate the problem, review possible solutions, and agree on a way forward.

**Figure 2.**
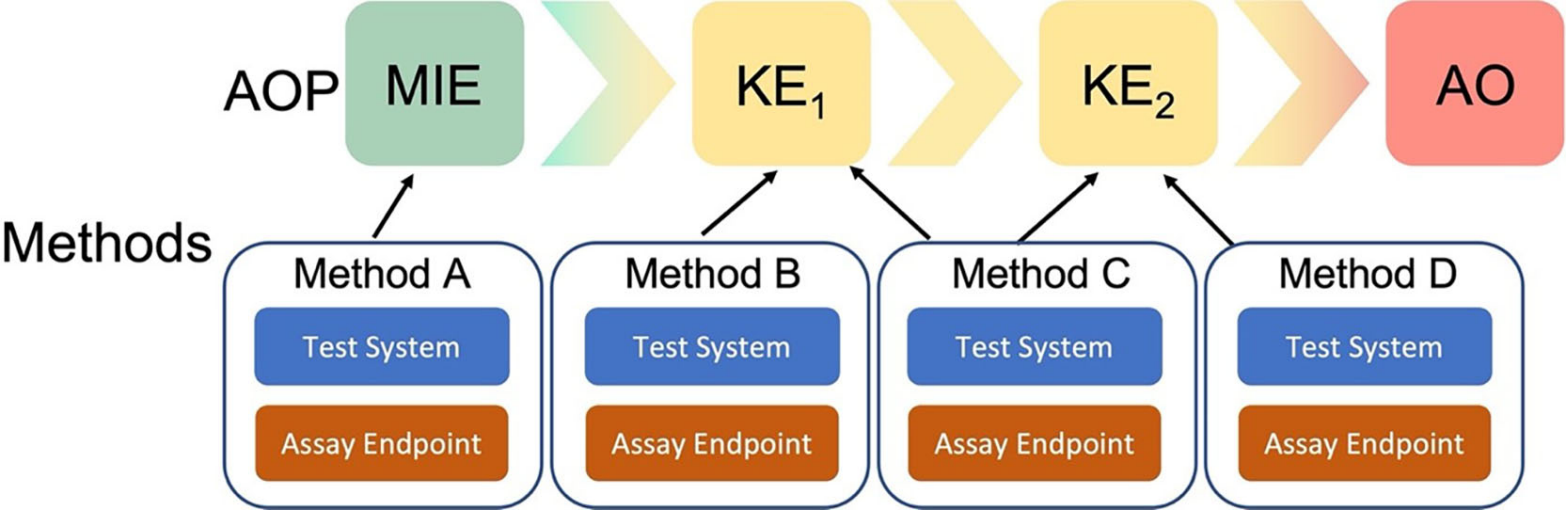
Methods information incorporated to AOPs. The Methods2AOP collaboration proposes the integration of discrete methods information into the Adverse Outcome Pathway (AOP) framework where the AOP construct portrays existing knowledge concerning the linkage between a molecular initiating event (MIE) and an adverse outcome (AO), via measurable key events (KE) at a biological level of organization relevant to risk assessment. The proposal allows for methods (visualized as A to D), including test system and assay endpoint, to be annotated to one or more KEs.

The Methods2AOP collaboration sought to refine the current “How It Is Measured or Detected” free text field into more structured documentation of methods information. Methods2AOP collaborators identified and defined relevant fields (including both the test system and the assay endpoint), and proposed a conceptual framework that would allow the association of methods information with one or more MIE/KE(s)/AO ( Figure 2). More specifically, the proposal includes detailed, structured annotation of methods that could benefit AOP-Wiki users as well as the method developer community. Inclusion of method developers takes the burden off the AOP developers and opens the door for further broadening the AOP community and facilitating collaborations. The envisioned outcome of implementing the Methods2AOP collaboration’s proposal would allow for interoperable methods information across the AOP-Wiki reinforcing the biological relevance of a NAM, thus helping build confidence in NAMs and AOPs for regulatory application.

The Methods2AOP collaboration started as a discussion group of a few like-minded individuals which quickly attracted international interest. The original crowdsourcing approach that drew in stakeholders from the existing AOP community quickly evolved into a collaboration of people who represent diverse viewpoints and backgrounds, all committed to integrate the latest state of the science and policy into the AOP community. Acknowledging existing related activities focused on assay description and documentation ( Krebs et al., 2020; Krebs et al., 2019; Bal-Price et al., 2018; Leite et al., 2024), the Methods2AOP collaboration established strong connections to parties that had embarked on similar efforts, including some joining the collaboration outright. It was important that the Methods2AOP collaboration engaged government, academia, industry, and contract research organizations who all have experience with assay development and use from different perspectives. Regulators, researchers, commercial users, and industry end users with familiarity of using AOPs or NAMs were engaged to create the collaboration. This diversity was paramount to work toward attracting a new demographic into the AOP community: test method developers.

### 2.2 Identifying and prioritizing relevant methods information

The Methods2AOP collaboration defined scope, confirmed relevance, and found balance in identifying and prioritizing sufficient and appropriate fields to document methods information. The initial scoping process identified several potential fields and revealed initial major challenges such as determining if a field should be required, assessing its usage potential (e.g., would AOP authors or regulators find this field informative and use it), and linking fields with an appropriate structured controlled vocabulary/same language. To exercise restraint and not overcomplicate, discussions were focused on the minimum critical information for AOP use and relevance to AOP adoption for regulatory applications. With this in mind, the collaboration prioritized key information by leveraging group polling to integrate ranking preference across the Method2AOP members.

Votes of variable weight were awarded such that each voter was required to award points to each field/contestant (8 points for the most favorite field, 1 point for the least favorite). In the end, fields with the highest number of points ranked highest. The outcome of this attempt identified 24 fields relevant to methods information that then were grouped based on their “scope”: (1) scientific/technical, (2) analysis/interpretation and (3) validation/regulatory ( Table 1). This exercise showed that more defined, discrete fields were needed. Interestingly, the highest ranked fields were the most defined while the open-ended information fields were among the lowest-ranked ones. Thus, by polling collaboration members, important information regarding not only scientific content but also what makes a field informative was gathered. These fields were then evaluated by populating them with real methods information from specific KE chosen by various members of the Methods2AOP collaboration.

**Table 1.** Information fields initially scoped and ranked by the Methods2Aop collaboration.

Rank	Field scope	Information documented	Input type
1	Scientific/Technical	Targeted biological endpoints (process or pathway/gene or protein/metabolites)	ConVocab
2	Analysis/Interpretation	Relationship between detection technology and measured endpoint	ConVocab
3	Validation/Regulatory	Measured observation (if relationship is indirect)	ConVocab
4	Scientific/Technical	Method type	ConVocab
5	Validation/Regulatory	Standard protocol availability	ConVocab
6	Scientific/Technical	Experimental setting	ConVocab
7	Scientific/Technical	Detection technology	ConVocab
8	Scientific/Technical	Species specificity	ConVocab
9	Validation/Regulatory	Method trade name and provider	ConVocab
10	Scientific/Technical	References	ConVocab
11	Scientific/Technical	Tissue or cell type specificity	ConVocab
12	Validation/Regulatory	Status of the method (validated?)	ConVocab
13	Analysis/Interpretation	Relevance to the AOP KE (one per linked KE)	ConVocab
14	Analysis/Interpretation	Other KE this method has been applied to	ConVocab
15	Scientific/Technical	Interferences or confounder	Free Text
16	Scientific/Technical	Limitation(s)	Free Text
17	Scientific/Technical	Summary	Free Text
18	Scientific/Technical	Duration of treatment	ConVocab
19	Scientific/Technical	Window of sensitivity	ConVocab
20	Scientific/Technical	Complexity level	ConVocab
21	Analysis/Interpretation	Preference ranking among methods for this KE	ConVocab
22	Validation/Regulatory	Popularity	Free Text
23	Scientific/Technical	Other factors to be considered	Free Text
24	Scientific/Technical	Factor to be considered for IVIVE applicability	Free Text

Note: Fields were ranked by 14 members of the Methods 2AOP collaboration.

ConVocab: Controlled vocabulary/same language ^8^ (entry would be defined by a term list, taxonomy, or ontology).

### 2.3 Organizing and standardizing relevant methods information

**Figure 3.**
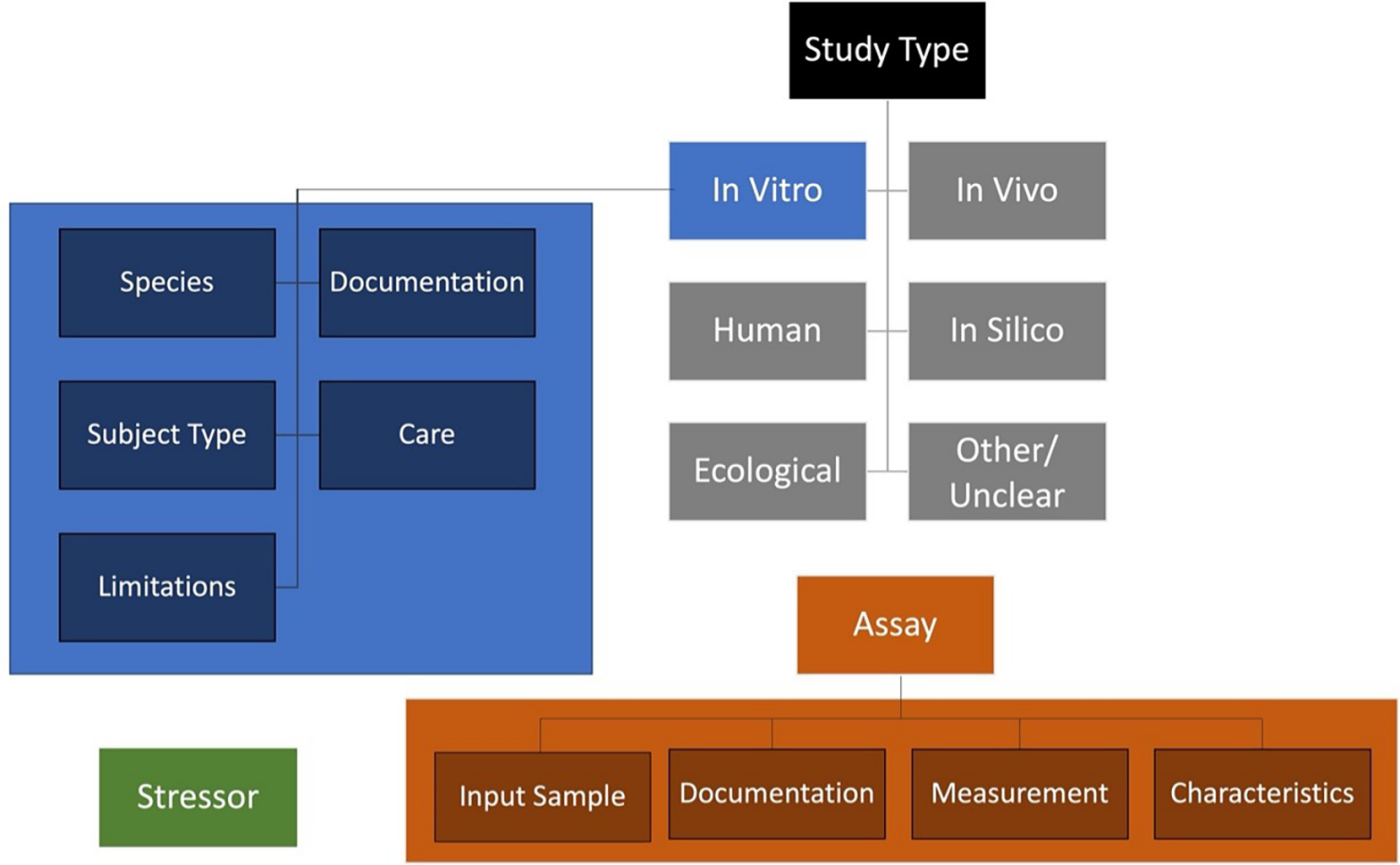
Method information tiering strategy. Discrete Study Types are listed, each of which would have study type specific methods and detailed information. The study types include *in vitro*, *in silico*, *in vivo*, human, ecological, and other/unclear. Specific methods information groups for the *in vitro* study type are shown in blue (detailed fields are provided in Figure 4). In addition to the study type information, stressor information would be independently defined (green), as would assay information pertaining to technical aspects of quantification/readouts (orange). In sum, all this information would be combined in a comprehensive document relevant to interpreting methods data in the AOP context (these relationships are defined conceptually in Figure 5).

To elevate the description of test methods from free text to structured information that is more easily searchable and comparable, metadata field organization was refined and ontologies were introduced. Content fields were organized hierarchically into tiers to offer further context and structure ( Figure 3). Each of the content tiers contains numerous fields of detailed information for which ontological corresponding terms were identified, where available, from the Open Biological and Biomedical Ontologies (OBO) Foundry ^9^ ( Karmaus et al., 2025). Establishing some standardized fields that leverage controlled vocabularies to ensure alignment with the FAIR principles (findable, accessible, interoperable, and reusable). ^10^ A simple structure of tiers for method information was defined: (1) study type, (2) study type specific methods information groups, (3) detailed fields per group. The study types include *in vitro*, *in silico*, *in vivo*, human, ecological, and other/unclear (e.g., *ex vivo* where likely a complement of *in vivo* and *in vitro* fields could be required to document sample origin in addition to subsequent methodology). Each of these study types would have their own groups of methods information needs.

**Figure 4.**
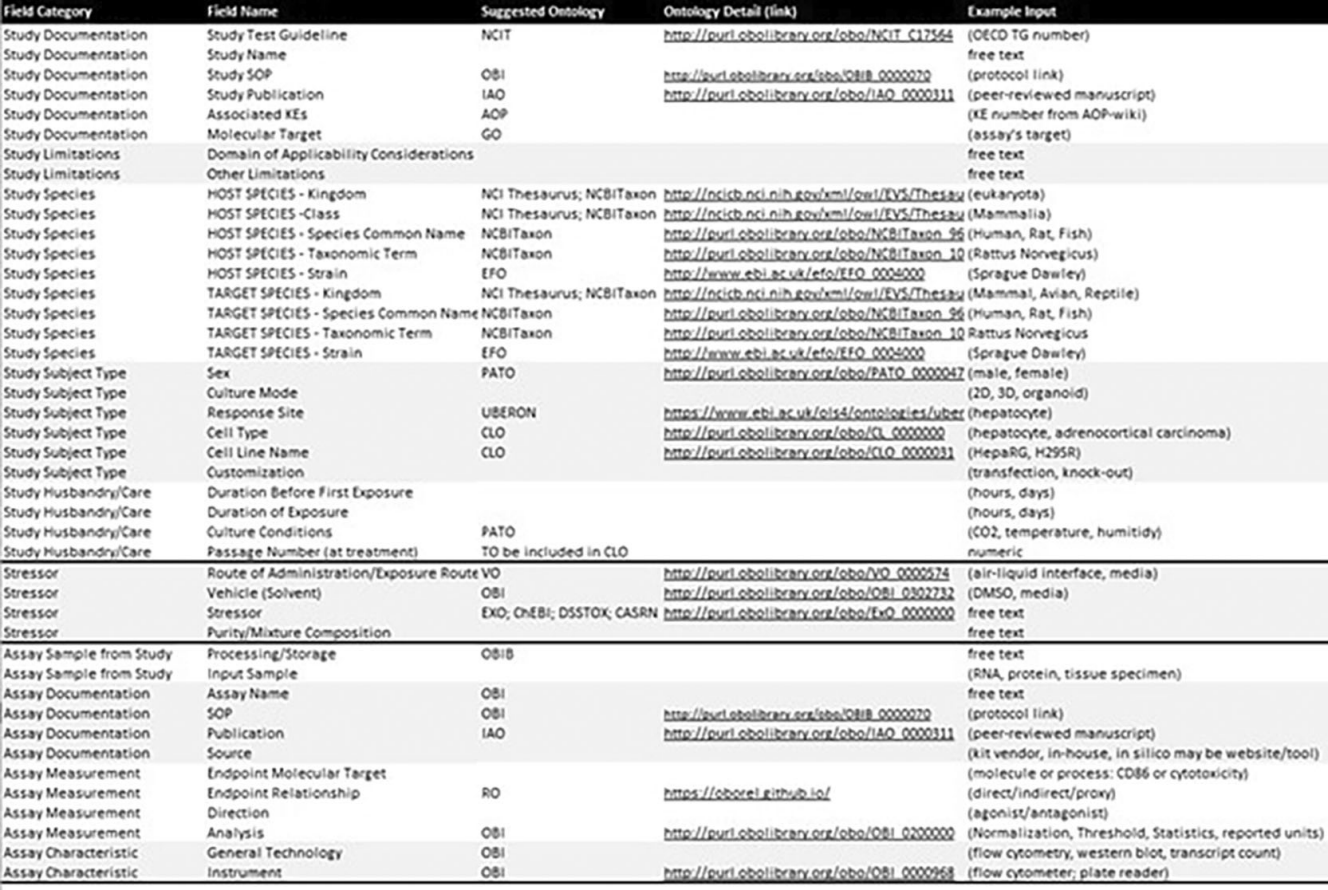
Example of the detailed fields within the in vitro Study type for methods integration to the AOP infrastructure. Stressor detailed fields and Assay detailed fields are also provided to offer a more complete representation of information that would be documented from the new tiered structured data approach.

As an example, the details relevant to the *in vitro* study type are presented in Figure 4 including documentation, species, subject type, care, applicability domain, and input sample/assay. To conceptually structure test method information within the context of the existing AOP-KB Data Model, the Methods2AOP collaboration proposes an interactive progressive disclosure interface that would prompt appropriate content input presenting only the necessary or requested information to users at any given time, thereby keeping the interface simple and clean. This approach would gradually reveal additional options, features, or content as needed (e.g., select a study type and then see the fields relevant to that study type in the next step). Employing a progressive disclosure approach for information submission reduces the cognitive burden placed on content submitters, while facilitating an opportunity for deeper engagement and comprehension of details for AOP-Wiki information consumers.

The pivotal role of ontologies in this refined approach cannot be overstated. Showcasing real-world application of ontologies to replace formerly free-text fields underscores how data standardization can be leveraged to update the AOP framework, facilitating easier navigation, input, review, and interpretation for ultimate use. The selection of ontologies was guided by their consistency and the ease with which they facilitate the integration of terms ( Figure 4). The use of ontologies provides a structured framework that ensures terms are aligned and interoperable within the broader data ecosystem. By establishing clear relationships with the annotation terms used, we elevated the description of test methods from basic textual representations to comprehensive and meaningful structured sets of methods information. Furthermore, this can support identifying overlaps where methods can inform on more than one KE, drive the adoption of robust methods to assess KEs, and build trust and confidence from the regulatory community toward accepting data from methods selected to inform on steps in any given AOP.

## 3. Prospective integration and implementation

**Figure 5.**
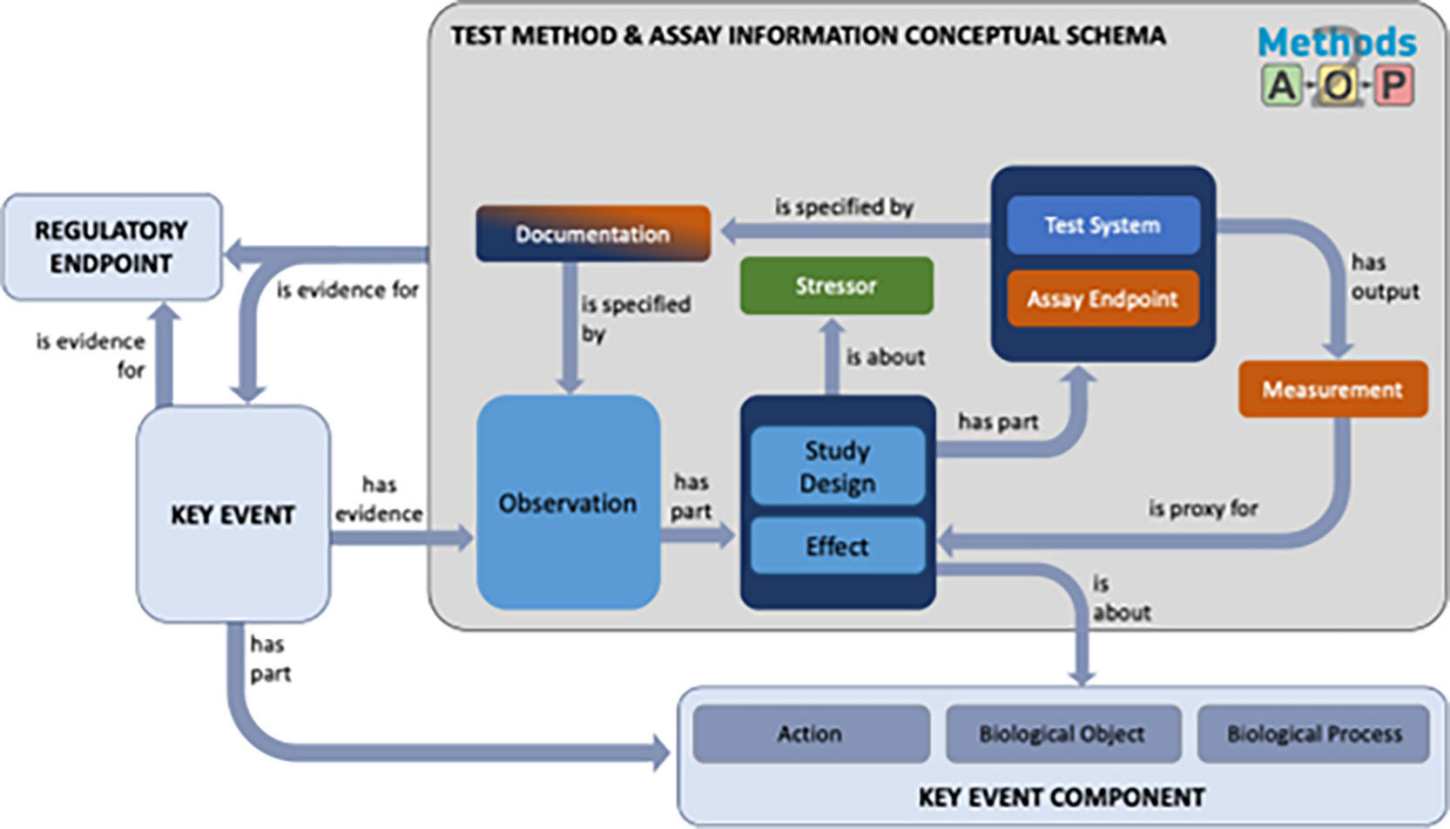
Methods2AOP test method and assay information conceptual model. Schematic diagram demonstrating the contributions outlined by the Methods2AOP collaboration (within the large dark grey box) and its connection points to existing AOP-KB components in light blue (e.g., Regulatory Endpoint, Key Event, and Key Event Component. Terms are linked by defined relationships that each serve a distinct purpose within the schema determined by their ontological definitions and the nature of the entities they connect. Relationship definitions: “is proxy for” (ontological reference AFX: 0002821) representing a surrogate entity used for practical measurement or observation when the actual target entity cannot be directly referenced. This is particularly important in our schema where measurements serve as proxies for underlying biological phenomena. “Has evidence” / “is evidence for” (RO: 0002558/RO: 0002472) captures the support relationship between an observation (or material/process) and the assertion it substantiates. These relations articulate how test method components provide grounding for Key Events or Regulatory Endpoints. “Has part” (BFO: 0000051) reflects the compositional relationship, correctly applied where an entity (e.g., Observation) structurally includes subordinate components (e.g., Study Design, Effect). “Is specified by” (SIO: 000339) relates an entity to an information content entity that describes or specifies it. “Has output” (RO: 0002234) relates a process and an entity (material or information) generated as a result of that process. “Is about” (IAO: 0000136) a relationship between an information content entity and the entity it is about.

The Methods2AOP collaboration further discussed how methods information prospectively could be integrated into the current AOP-KB framework. In the current AOP-Wiki infrastructure KEs that are AOs have an input field for entering the “Regulatory Significance of the Adverse Outcome”, thereby providing a way to document linkages between regulatory endpoints and AOPs. Moreover, KEs contain key event components, comprised of a biological object and/or process, along with an action term that describes a direction of perturbation ( Ives et al., 2017). We propose a conceptual model that highlights the qualitative similarities between concepts used in key event components and the structured and detailed methods information suggested by the Methods2AOP (illustrated by the grey box in Figure 5).

KEs do not currently have a field for “Observations” in the AOP-KB, rather there is a “methods of measurement” free text box. The proposed conceptual model, includes an “observation” field defined as comprising information on study design and the measured effects (for which detailed inputs are summarized by the tiered data and detailed fields in Figures 3 & 4). “Observation” is not currently part of the AOP-KB data model, however, its preliminary integration is already under development and has been presented as part of previous KER-centric Evidence Modeling prototyping efforts ( Hench et al., 2024a; O’Brien and Yauk, 2022).

The Methods2AOP collaboration conceptual schema leverages structured relationships to ensure connections between information. The conceptual schema would be part of the Key Event as supporting evidence and is broken in to key elements wherein the “observation” has parts (is associated with information about) “study design” and the “effect” being evaluated. The “effect” would be directly about (and linked back to) the existing Key Event Component currently defined in the AOP-KB. Further attributes defined in the schema that are associated to these fields include “stressor” (e.g., test article), information about the “test system” and “assay endpoint”, and attributes providing “documentation” and the output “measurement” (fields that would comprise study design and assay details Figures 3 & 4). Figure 5 contains these terms and shows the relationship between the information. Type, details, and structure of information that could be added to the AOP-KB regarding study design and assay are described in more detail in Figure 4.

Integrating more detailed methods information could strengthen the confidence of the association between the method and the KE and consequently support the evidence for the regulatory endpoint. The value of ontological approaches lies in its ability to decompose all the information associated with a method and then reconnect them by utilizing specific defined relationships. In this ontological representation, the term “method” is not synonymous with “assay”; rather, it encompasses a broader set of information, including the study design and technical assay itself as well as the additional context, as illustrated in Figure 5. This comprehensive approach ensures that all aspects of the method are documented and interconnected. The value of the ontological approaches to information organization is exemplified by the relationships/associations that are defined on each arrow. By establishing relationships to help make connections among the structured data, more information can be stored in the AOP-KB in a manner that is both computable and useful for humans.

## 4. Stakeholder engagement

Subsequent to identifying, prioritizing, and organizing relevant methods information, the Methods2AOP collaboration actively engaged with, and sought feedback from, various stakeholders. Through presentating at various international conferences and hosting a focused workshop, broad feedback was collected. For example, stakeholders from the regulatory or government sectors were invited to participate in a virtual workshop entitled “Roles of AOP framework in incorporating NAMs into the regulatory process” in April 2024. Presentations at this workshop described how AOPs could support the use of NAMs in regulatory decisions and introduced the ongoing work by the Methods2AOP collaboration to improve the methods documentation in the AOP framework.

These presentations were followed by breakout group discussions which yielded feedback on how the various participants currently used the AOP framework and NAMs, how the suggested inclusion of the methods information could influence the use of AOPs and NAMs, and what their perceived future needs and wishes were. Three individual questions were posed to participants; the key themes discussed for each question are shown in Table 2. Some of the common themes proposed for facilitating the regulatory acceptance were already part of the Methods2AOP collaboration, e.g., establish more explicit linkages of AOPs to the test methods used in MIEs/KEs, make validation status of methods used to measure KEs and develop AOPs immediately visible and transparent, etc.

**Table 2.** Charge questions and themes discussed in the workshop.

#	Questions	Themes discussed
1	Describe scenarios where use of the AOP framework (assays connected to key events and key events to AOs) could make your workflow faster, easier, more transparent, or better in some other way	• Better understanding which specific methods were used to support MIEs and KEs • Linking methods to KEs could bring confidence to both the method and the AOP • Help identify opportunities for read across, improve read across or to propose data that can support a waiver for in vivo studies • Support the design of NAMs batteries or IATAs • Identify orthogonal methods and help to build confidence that a KE is really part of the mechanism that causes the AO • OECD endorsed AOPs implies that the mechanistic links are more reliable than for non-endorsed
2	What information or resources would you need to feel confident in the methods/assays and their connections to KEs to be able to incorporate the AOP framework into your existing workflows?	• Documentation for both the method and method linkage to the KE are critical in establishing trust in the AOP and the relationship between Method/KE and KE/AO • Information on method uncertainty to evaluate the evidence for KEs • Validation or readiness status of a method is important to build confidence in that the data is useful for regulatory applications • Quantitative aspects, e.g., what does a 10% alteration in a KE mean • Inclusion of KERs in the AOP framework is crucial • Need agreement on what we are validating the AOP against since human data is limited
3	Based on the proposal from the Methods2AOP, what information is needed to best support workflow needs to better use NAMs? In an ideal world, what would you like to see in the AOP-Wiki?	• Development of AOP networks to support regulatory applications • Early engagements by regulators in AOP development, to enhance their use in regulatory applications • Evaluate the relevance of KEs withing the AOP, e.g., are there a “point of no return”, are some KEs more relevant than others for the AO? • Document the timeline of AOP endorsement, progress and when it is used • Ability to link to open databases, possible literatures to help gain information of the AOP and methods • Revise the review of AOPs to make the process easier and faster

Three newly proposed common themes were identified by workshop participants representing potential opportunities for future initiatives, implementations, and pilot application cases on specific disease adverse outcomes (e.g., liver injury, neurotoxicity, carcinogenicity). These three common themes were:

1. Identification of reference chemicals/causal agents;
2. Identification of inter-related AOPs (AOP networks), and inclusion of more information within AOPs for the relationship between KEs;
3. Evaluation of the usefulness of quantifiable data/points of departure (POD)/no observed effect concentrations (NOECs) in concentration-responses.

Based on the number of registrants and the captivating and constructive discussions achieved during this virtual workshop, similar events will be planned in the future. This will ensure stakeholder engagement with an open and active channel of communication between all parties involved.

## 5. Key takeaways and impact

The main purpose of the Methods2AOP collaboration is to increase confidence in AOP knowledge and boost its uptake in being leveraged for data generation by better structuring the information of the methods used to characterise KEs and KERs. Ultimately this could enhance the acceptance of AOPs and NAMs for regulatory applications. There are regulatory processes (e.g., European Food Safety Authority (EFSA) Pesticides Peer Review) where academic publications are reviewed and taken into consideration; however, before data are accepted, they need to be considered reliable and relevant. This includes a step of assessing the risk of bias of the study, which is only possible when it is described with enough detail regarding the method, data interpretation and conclusions ( EFSA, 2010). Unfortunately, this aspect is often overlooked by the authors of such studies, limiting the impact of the work. To overcome such potential limitations within the AOP context, further efforts need to focus on developing standardized methods reporting and provide consistency in data interpretation, as this would bring more visibility to how approaches are performed and how they can inform on KEs.

The Methods2AOP collaboration worked to find balance in identifying and prioritizing sufficient and appropriate fields to document methods information. Focused on end-users in the AOP community, the Methods2AOP approach not only enhanced the clarity of method descriptions and proposed a tiered data structure; the Methods2AOP collaboration also engaged stakeholders to ensure needs could be met and the solutions proposed are of relevance. To overcome ambiguity of free-text fields and support standardized information structure to increase functionality and interpretability, we propose the establishment of standardized fields that leverage controlled vocabularies/ontologies alongside existing free-text options. This hybrid model promotes enhanced interoperability across AOPs, enabling the seamless reuse of methods and the identification of overlaps where a single method may inform on multiple KEs and AOPs. Such standardization could not only foster the adoption of robust methodologies for assessing KEs but also cultivate trust and confidence within the regulatory community and be applied across the entire AOP-Wiki (i.e., not only for methods information refinement). By ensuring that data are generated with fit-for-purpose design to support investigation of the AOP, integration of method documentation makes it easier to input, review, and search methods information as well as assess data generated by them.

The application of robust methods documentation not only fulfills the role of addressing regulatory requirements but also empowers industry scientists and AOP users seeking to leverage AOP-based lines of evidence to assist mechanistic investigation. AOP authors, and reviewers, must provide sufficient information to meet AOP users’ needs for AOP knowledge to be applicable in practice, regulatory or research. The academic research community may use AOPs in a different context than risk assessors and regulatory scientists who are seeking established mechanistic lines of evidence. For example, detailed methods information can serve as a resource beyond the AOP context to assist scientists in designing their experiments or interpet their data.

Furthermore, having methods information structure that separates assay details from study design also opens the door to increase AOP community membership and encourage method developers to engage and contribute appropriate details for robust documentation. Engaging test method developers will provide an opportunity to promote relevant test methods and/or to scan for “underserved” KEs (i.e., KEs that have been defined but for which a suitable test method has not yet been sufficiently developed or validated). Such a win-win scenario would both highlight new opportunities for test method developers and help fill knowledge gaps in the AOP-Wiki.

## 6. Challenges and future needs

Several key challenges were identified by the Methods2AOP collaboration and will likely be encountered if changes are made to the existing AOP-Wiki content to integrate the data structure and detailed documentation proposed herein. The Methods2AOP collaboration focused on engaging a diverse consortium, identifying relevant metadata, organizing, annotating, ontologizing fields, and devising a proposed vision for implementation. Outstanding themes discussed by the collaboration but not yet addressed include addressing how ease of use would be paramount to the adoption of any redefined methods details. Easing accessibility (e.g., via the simple tiered data input proposal) and committing to education for both input and retrieval of methods information, including revison of the AOPs Developer’s handbook, will be critical to the successful implementation, impact, sustainability, and growth of the AOP community. AOP authors and methods developers will need training to fill out the right information and the benefit of doing so will need to be well communicated to ensure participation and engagement.

This is similar to the continuing need for guidance for method developers embarking on validation studies and Test Guideline Development of new assays for use in the international regulatory context (OECD Webinar Series). ^11^ Although information fields for methods have been identified and suggested here, the Methods2AOP collaboration members recognize the need to develop case studies to evaluate and likely refine required information to ensure an easy and user-friendly approach and establish applicability to various contexts of use. In this context, taking a cue from the standard fields identified in OECD Test Guidelines (e.g., OECD GD211-describing non-guideline *in vitro* test methods), ^12^ and OECD Harmonized Templates ( Carnesecchi et al., 2023) (e.g., OHT201), ^13^ may provide additional information on the way forward in the future progress of Methods2AOP.

In addition, prospective changes made to the AOP-KB and interface of the AOP-Wiki would require considerable effort and restructuring that will ultimately impact a variety of third-party tools. Thus, Methods2AOP seeks to suggest content, but not to conduct the implementation of this information at this stage. While the proposed revision of the current AOP-KB structure and Data Model relationships could be substantial upon integration of methods information, it will be a benefit that all tools draw from this centralized resource. If designed carefully, third-party tools could retain backward compatibility and be updated to include the new information in their own schedule. Future efforts should include alignment of AOP mechanistic data in collaboration with other programs that aim to improve FAIR standards such as the FAIR AOP Cluster working group ( Mortensen et al., 2025), the Environmental Health Language Collaborative (EHLC), ^14^ and the ELIXIR Toxicology Community workgroup. ^15^ Finally, continued engagement with stakeholders to provide resources and education about any changes and updates would be critical for seamless uptake. To summarize, the work executed by the Methods2AOP collaboration could benefit a diverse group of stakeholders in different ways with tangible foreseeable impacts ( Table 3).

**Table 3.** Stakeholder groups that can benefit from the inclusion of structrued methods information in the AOP framework.

Stakeholder group	Next steps	Potential benefits
AOP Framework Management at OECD	• Understand the benefits of a methods centered approach and appreciate the relevant Methods2AOP suggestions • Facilitate, support, and promote the adoption of Methods2AOP insights in the AOP Framework • Assist in the identification of possible funding sources to implement the Methods2AOP recommendations in the AOP-KB/Wiki	A more widely adopted and powerful AOP concept
AOP-KB/Wiki Funding Bodies	• Understand the impact that Methods2AOP recommendations can have on the regulatory landscape • Acknowledge how a methods-centered AOP framework gains visibility and boosts market opportunities for an adequate ICT solution • Actively encourage ICT developers to submit proposals for a Methods2AOP-powered next version of the AOP-KB/Wiki	Increased return on investment in an improved AOP-Wiki
AOP-KB/Wiki ICT Developers	• Understand how the Methods2AOP recommendations would translate into ICT requirements • Identify gaps between the current AOP-KB/Wiki situation and these requirements • Help other stakeholders (e.g. funders) to understand potential costs and prioritize various options and make a convincing case for implementation	Enhanced technical knowledge to support ICT development
Regulators (not directly involved in AOP application)	• Facilitate the integration of AOPs for regulatory decisions • Bring case studies for a more methods-oriented AOP approach to bodies like OECD, etc. • Support AOP users within their jurisdictions in the phase-in of the new approach by empowering them to take decisions based on Methods2AOP-powered AOP knowledge	Benefit from a pioneering/early adopter role in the introduction of a methods-centered paradigm More tools to support regulatory decisions
AOP Users (including industry, academic, and regulatory stakeholders directly involved in AOP application)	• Understand the way more tangible methods-AOP connections can increase their decision quality • Support, from a hands-on perspective, the introduction of a methods-oriented approach among peers • Have more confidence leveraging AOP knowledge	More robust data and higher confidence in using AOPs and mechanistic assay based lines of evidence to support decisions
AOP Developers/Authors	• Understand the way minor extra effort (i.e. add methods information to their AOPs) can boost their AOP’s usefulness in regulatory circles • Show support for the paradigm change in gatekeeper circles • Adapt own AOP development strategy accordingly	Increased visibility and scientific/regulatory impact of their work
Method Developers/Vendors	• Familiarize and have more insight to regulatory applications • Promote the method-centered AOP approach in the method developer community • Identify gaps for future assay development opportunities	Increased visibility and broader use of products
Animal Advocacy Groups	• Understand the important role an increased focus on methods in the AOP framework can have on regulatory adoption of NAMs • Informed to discuss the Methods2AOP suggestions with policy makers • Support the paradigm change in replacing animal testing	Faster and more-widespread adoption of NAMs to replace animal use

ICT: Information, and Communication Technology.

## Disclaimer

This manuscript does not necessarily represent US Environmental Protection Agency (US EPA) policy or any other US agency. This manuscript has been subjected to review and approved for publication by the US EPA’s Office of Chemical Safety and Pollution Prevention. Mention of trade names or commercial products does not indicate endorsement by the US EPA. This manuscript was not reviewed by and does not reflect the view of 3M.

## Data Availability

The original contributions presented in the study are all contained in the article Figures and Tables. In addition, the study refers to the following underlying data, which is a table providing the extended data used to support the analyses described in Section 2.3. The table is available here: https://doi.org/10.5281/zenodo.17650865, under the terms of the Creative Commons Attribution 4.0 International license (CC-BY 4.0). Further inquiries can be directed to the corresponding author ( Karmaus, A., Bisson, W., Fostel, J., Masci, A. M., Wittwehr, C., & Hogberg, H. (2025).)

## Footnotes

^1^

https://aopwiki.org/info_pages/10 [accessed May 2, 2025]

^2^

https://aopwiki.org [accessed April 17, 2025]

^3^

https://aopkb.oecd.org/index.html [accessed April 17, 2025]

^4^

https://www.niehs.nih.gov/research/programs/ehlc/events [accessed May 5, 2025]

^5^

https://elixir-europe.org/communities/toxicology [accessed April 17, 2025]

^6^

https://one.oecd.org/document/ENV/JM/MONO(2013)6/en/pdf [accessed April 17, 2025]

^7^

https://aopwiki.org/handbooks/5 [accessed April 17, 2025]

^8^

https://www.niehs.nih.gov/research/programs/ehlc/shared-language [accessed April 18, 2025]

^9^

http://obofoundry.org [accessed April 18, 2025]

^10^

http://go-fair.org [accessed April 18, 2025]

^11^

https://www.oecd.org/en/topics/sub-issues/testing-of-chemicals/webinar-series-on-testing-and-assessment-methodologies.html [accessed April 17, 2025]

^12^

https://www.oecd.org/en/publications/guidance-document-for-describing-non-guideline-in-vitro-test-methods_9789264274730-en.html [accessed April 17, 2025]

^13^

https://www.oecd.org/en/topics/assessment-of-chemicals/harmonised-templates-intermediate-effects.html [accessed April 17, 2025]

^14^

https://www.niehs.nih.gov/research/programs/ehlc [accessed April 17, 2025]

^15^

https://elixir-europe.org/communities/toxicology [accessed April 17, 2025]
