# Supplementary material for: Methods2AOP: A Collaboration to Strengthen the Integration of Test Methods into the Adverse Outcome Pathway Framework
Source: F1000Res. 2026 Feb 18;14:1375. Originally published 2025 Dec 8. [Version 2] doi: 10.12688/f1000research.172881.2 (PMC13151919; doi:10.12688/f1000research.172881.2)
Supplement: Peer review history

# Reviewer report: Reviewer response for version 2

**DOI:** 10.5256/f1000research.195945.r462675

**Published:** 2026-03-24

**Authors:** Olga Tcheremenskaia

**Affiliations:** 1 Insituto Superiore di Sanità (ISS), Rome, Lazio, Italy

**General comments**

 The manuscript analyses how the current free-text field “How It Is Measured or Detected,” used to describe Key Events (KEs) in the AOP-Wiki, could be refined into a more structured documentation format for methods information. This is highly valuable work for both the scientific and regulatory communities, as it directly addresses current limitations in transparency, interoperability, and regulatory uptake of methods information within the AOP framework.

 I fully agree with the comments made by the two previous reviewers and with the revisions implemented by the authors. The supplementary materials are particularly useful, as they provide detailed examples of how selected *in vitro*, *in vivo*, and *in chemico* methods can be mapped to ontology fields. The authors have done an excellent job identifying and reusing 15 existing ontologies (e.g. OBI, GO, ChEBI), in line with established best practices for semantic interoperability and the FAIR principles.

 Overall, the work is timely, well-motivated, and highly relevant to the further integration of New Approach Methodologies (NAMs) into regulatory decision-making via the AOP framework.

**Major comments**

**1. Mapping to OHT (for instance hCLAT to OHT 66‑1)**

 While the supplementary material provides excellent practical examples, such as the human Cell Line Activation Test (hCLAT) annotated using externally identified ontology fields, I would ecommend that the authors additionally map this example (or other two skin sensitisation related examples) directly to OECD Harmonised Template (OHT) 66‑1 (Skin Sensitisation). OHT 66‑1 is an established endpoint‑oriented template that has been updated to accommodate non‑apical observations and to link these to Adverse Outcome Pathways (AOPs)

 I consider it important to evaluate how the proposed Methods2AOP fields align with the structured fields of OHT 66‑1. Such a mapping would provide a clear regulatory pathway and facilitate the direct use of Methods2AOP‑annotated methods in regulatory dossiers and Defined Approaches on skin sensitisation.

**2. OHT terminology versus external ontologies**

 The use of external ontologies is highly useful and clearly strengthens semantic interoperability. However, it may be helpful first to evaluate how existing endpoint‑oriented OHT templates (which are not exclusively mechanistic, unlike OHT 201) already accommodate test system, assay, and observation descriptions. A key advantage is that OHTs include suggested terminology and picklists that are already familiar to the regulatory community.

 For example, for cell lines “Cell line name: THP‑1 (CLO:0009348)” in OHT 66‑1, field number 44, the picklist entries such as: and “Details of test system: THP‑1 cell line [442E]; keratinocytes transgenic [442D]; THP‑G8 [442E]; U‑937 [442E]; other.”

 Leveraging OHT terminology and picklists could help make test system and assay descriptions more readily usable in Defined Approaches (DAs), Test Guidelines (TGs), and validation studies conducted in line with OECD Guidance Document 34 (GD34). Looking forward, as additional OHTs are updated to capture non‑apical endpoints, it would be beneficial if the Methods2AOP framework could always foresee mapping to OHT terminology where possible.

**Minor comments and suggestions**

**1. Glossary of core terms**

 To improve clarity, the manuscript would benefit from a short glossary that explicitly defines the core terms used throughout the Methods2AOP framework. In particular, it would be helpful to clearly distinguish between:

- Test System (the biological model),
- Assay (the specific technical process), and
- Observation (the composite entity encompassing study design and measured effects).

Although these definitions are available in the supplemental file “Suggested Ontologies and Fields – Term Definition,” briefly summarizing them in the main text would enhance accessibility for readers less familiar with the terminology.​

**2. Feasibility of revising existing AOPs**

 I agree with the authors that refining the current “How It Is Measured or Detected” free-text field into structured documentation will be a resource‑intensive process. This will likely require revising the AOP Developers’ Handbook and updating the guidance for AOP Coaches. It would be useful if the authors could explicitly discuss whether the proposed approach is intended only for newly developed AOPs or there is a strategy to address the existing AOPs in AOP‑Wiki, some of which remain incomplete, outdated, and not yet endorsed.

**3. Potential use of large language models (LLMs)**

 Given the considerable manual curation effort required, it might be worth briefly exploring the potential use of large language models (LLMs) to support and speed up the standardization of the existing free‑text “How It Is Measured or Detected” fields, under appropriate human oversight. A short discussion if the Methods2AOP structure could be used as a target schema for semi‑automated text-to-structure mapping would be of interest to the community.

**4. Field for the linked Key Events**

 Although Key Event Relationships (KERs) are not the primary focus of this work, the authors could consider introducing a dedicated field, where to indicate if the KE is linked to another KE.

**5. Possibility of prioritization of methods approaching regulatory uptake**

 The manuscript rightly notes that any future changes to the AOP‑KB and the AOP‑Wiki interface would require considerable effort and would affect multiple third‑party tools. In the short term, it might therefore be more feasible to keep the current free-text field in the AOP‑Wiki but provide concise, practical guidance on how to standardize this free-text when a test is intended for regulatory purposes, such as Defined Approaches (DAs) or for evaluation under GD34 as a Test Guideline candidate. Once the ongoing update of GD34 is finalized, the Methods2AOP information could potentially be mapped directly to the readiness‑criteria templates in GD34, thereby helping to bridge the gap between AOP development, method documentation, and formal regulatory validation.

Is the work clearly and accurately presented and does it cite the current literature?

Yes

If applicable, is the statistical analysis and its interpretation appropriate?

Not applicable

Are all the source data underlying the results available to ensure full reproducibility?

No source data required

Is the study design appropriate and is the work technically sound?

Yes

Are the conclusions drawn adequately supported by the results?

Yes

Are sufficient details of methods and analysis provided to allow replication by others?

Yes

Reviewer Expertise:

Computational toxicology, regulatory toxicology, NAMs, databases

I confirm that I have read this submission and believe that I have an appropriate level of expertise to confirm that it is of an acceptable scientific standard.

# Reviewer report: Reviewer response for version 2

**DOI:** 10.5256/f1000research.195945.r464632

**Published:** 2026-03-10

**Authors:** Peijnenburg Willie

**Affiliations:** 1 National Institute for Public Health and Environment (RIVM), Bilthoven, The Netherlands

General assessment

 This manuscript presents the Methods2AOP initiative, a collaborative effort aimed at improving the integration of test method information (particularly New Approach Methods (NAMs)) within the Adverse Outcome Pathway (AOP) framework and its central knowledge repository, the AOP-Wiki. The authors argue that although AOPs provide a structured framework linking molecular initiating events and key biological processes to adverse outcomes, the documentation of experimental methods used to measure these events remains insufficiently structured. As currently implemented, AOP-Wiki entries often describe measurement approaches in a free-text field (“How it is measured or detected”), which limits interoperability, reproducibility, and regulatory usability of the underlying data. To address this limitation, the manuscript proposes a conceptual framework for structured documentation of test methods using standardized metadata fields and ontology-based annotations. The proposed Methods2AOP schema introduces structured entities such as “method,” “assay,” and “observation,” and organizes method information through hierarchical tiers and controlled vocabularies to facilitate FAIR (findable, accessible, interoperable, and reusable) data integration. The authors further outline how these structured descriptors could be linked to key events (KEs) within the AOP knowledgebase, thereby strengthening the connection between mechanistic toxicology data and regulatory decision-making frameworks.

 The topic is timely and highly relevant to the ongoing transition toward mechanism-based toxicology and the increasing use of NAMs in regulatory science. The manuscript is clearly written and provides a thorough overview of the collaborative effort, including stakeholder engagement, methodological prioritization of metadata fields, and conceptual implementation within the AOP knowledge infrastructure. Overall, the work represents a valuable conceptual contribution to the development of the AOP ecosystem. However, some aspects of the proposed framework would benefit from further clarification, particularly regarding practical implementation, conceptual boundaries between core entities, and long-term governance of ontology-based metadata.

 Major comments

 1 - Conceptual scope and intended role of structured “Observation” entities

 One of the central elements introduced in the proposed framework is the “Observation” entity, which is described as representing evidence derived from a test method and linked to key events within an AOP. The manuscript explains that this entity contains information about study design and measured effects, serving as supporting evidence for a KE. However, the practical scope of this entity remains somewhat ambiguous. It is not entirely clear whether the Observation entity is intended primarily as a metadata container describing how a KE is measured, or whether it is intended to represent potentially large numbers of individual study-level evidence instances generated across multiple studies and datasets. This distinction has important implications for database scale, curation burden, and long-term maintainability of the AOP knowledgebase.

 Clarifying whether the Observation concept represents a bounded metadata structure or a potentially extensible repository of study-level evidence would significantly strengthen the conceptual clarity of the framework. Additionally, it would be useful to explicitly discuss whether the framework is designed to capture contradictory or null findings, which are important for avoiding confirmation bias in evidence synthesis.

 2 - Conceptual boundaries between “method,” “assay,” and related entities

 The manuscript appropriately distinguishes between the broader concept of a “method” and the more specific technical “assay.” However, the practical distinctions between the entities used in the conceptual model (method, assay, observation, study design, and effect) are sometimes difficult to interpret when reading the text. While the conceptual diagram provided in the manuscript illustrates the relationships among these elements, a concise glossary or table defining each entity would significantly improve readability and conceptual clarity. In particular, readers would benefit from a practical example illustrating how information currently provided in the free-text “How it is measured or detected” field would map onto the proposed structured fields. Providing a concrete example demonstrating how a specific assay measuring a particular KE would be represented within the Methods2AOP schema would make the proposed framework more accessible and easier to evaluate.

 3 - Ontology use and long-term maintenance considerations

 The use of ontologies to standardize metadata and ensure interoperability represents one of the major strengths of the proposed framework. The manuscript highlights how ontology-based annotations can replace unstructured free-text descriptions and facilitate more robust data integration across different evidence streams. However, ontologies evolve over time as terms are updated, replaced, or deprecated. The manuscript briefly acknowledges the need for maintaining ontology versioning, but this issue could be discussed more explicitly. In particular, long-term traceability of annotations and compatibility with future ontology updates represent important challenges for any ontology-driven information infrastructure. A brief discussion of potential governance strategies—such as ontology version tracking, term mapping, or update mechanisms within the AOP knowledgebase—would strengthen the proposed framework and demonstrate awareness of these practical considerations.

 4 - Practical implementation and community adoption

 The manuscript appropriately emphasizes that the Methods2AOP collaboration proposes a conceptual schema rather than an immediate implementation within the AOP knowledgebase. Nonetheless, several aspects of real-world adoption remain relatively abstract. For example, implementing structured metadata fields within the AOP-Wiki would require substantial changes to the current database schema, user interface, and curation workflows. The manuscript acknowledges these challenges but could provide a more detailed discussion of possible implementation pathways, including pilot case studies or phased integration strategies.

 The authors note that early applications of the framework will likely represent an iterative learning process as contributors gain experience with the system. Explicitly framing the proposed approach as an evolving community-driven infrastructure could help set appropriate expectations for the adoption and refinement of the framework.

 Minor comments

 The manuscript is generally well written and logically structured. Nevertheless, several minor improvements could further enhance clarity.

 In the introduction, the discussion of the current AOP infrastructure could be slightly streamlined to focus more directly on the specific limitations that motivated the Methods2AOP initiative. Some sections describing stakeholder engagement and workshop outcomes could also be condensed without losing important content.

 Several figures provide useful conceptual illustrations of the proposed framework, particularly the diagrams illustrating the hierarchical structure of method metadata and the conceptual integration with the AOP knowledgebase. The captions could be slightly expanded to ensure that readers unfamiliar with the AOP framework can fully interpret the diagrams without consulting the main text.

 Finally, a short summary table describing the main components of the Methods2AOP schema would help readers quickly understand the proposed structure.

 Assessment of methodological transparency and reproducibility

 The manuscript presents a conceptual framework rather than an experimental study. Consequently, traditional criteria such as statistical analysis or experimental reproducibility are not directly applicable. Nevertheless, the authors provide sufficient detail regarding the development of the framework, including stakeholder consultations, prioritization of metadata fields, and ontology selection. The supplementary materials and linked datasets further support transparency of the proposed metadata structure. Overall, the methodological description is adequate to allow other researchers or infrastructure developers to evaluate and potentially implement similar approaches.

 Data availability and reproducibility

 The manuscript provides access to supplementary datasets containing extended metadata tables and ontology references through a publicly accessible repository. This is consistent with the open science principles promoted by the journal. The availability of these resources should allow readers to explore the proposed metadata structure and evaluate its potential implementation in other knowledge management systems.

 Conclusion

 This manuscript presents a well-conceived and timely proposal for improving the integration of test method metadata within the Adverse Outcome Pathway framework. By introducing structured, ontology-based descriptions of methods and linking them explicitly to key events, the Methods2AOP collaboration aims to enhance the transparency, interoperability, and regulatory relevance of AOP knowledge. The proposed framework represents a valuable conceptual contribution to the development of the AOP ecosystem and the broader transition toward mechanistic toxicology and NAM-based risk assessment. However, the manuscript would benefit from additional clarification regarding the conceptual scope of the Observation entity, clearer definitions of core entities within the proposed schema, and a more detailed discussion of practical implementation challenges. Addressing these points would further strengthen the manuscript and help readers better understand the potential impact and feasibility of the proposed framework.

 Final recommendation: approved with reservations

 The manuscript presents a valuable conceptual framework and contributes meaningfully to ongoing efforts to improve the interoperability and regulatory applicability of AOP-based toxicology. However, several aspects of the proposed structure would benefit from additional clarification and discussion before the framework can be fully evaluated by the community. With minor revisions addressing the issues outlined above, the manuscript will represent a useful and informative contribution to the literature on AOP infrastructure development and NAM integration.

Is the work clearly and accurately presented and does it cite the current literature?

Yes

If applicable, is the statistical analysis and its interpretation appropriate?

Partly

Are all the source data underlying the results available to ensure full reproducibility?

Yes

Is the study design appropriate and is the work technically sound?

Yes

Are the conclusions drawn adequately supported by the results?

Yes

Are sufficient details of methods and analysis provided to allow replication by others?

Yes

Reviewer Expertise:

AI, mechanistic toxicology, modelling

I confirm that I have read this submission and believe that I have an appropriate level of expertise to confirm that it is of an acceptable scientific standard, however I have significant reservations, as outlined above.

# Reviewer report: Reviewer response for version 2

**DOI:** 10.5256/f1000research.195945.r459591

**Published:** 2026-03-05

**Authors:** Alexandra Schaffert

**Affiliations:** 1 Tampere University, Tampere, Finland

Thank you for the careful revisions and for clarifying the previous comments. I appreciate the added worked examples in the supplementary material, the clearer discussion of ontology lifecycle/versioning, and the explicit acknowledgement that practical uptake of the framework will be an iterative learning process shaped by repeated real-world use. These additions make the proposal more concrete and strengthen the manuscript overall.

 My remaining comment is mainly conceptual, and I raise it because I think the current wording may still be difficult for readers to understand in relation to the existing AOP-Wiki structure.

 In the revised manuscript, the Methods2AOP proposal is still clearly framed as method-centric and KE-centric. The paper states that the intention is to “add to and refine the existing infrastructure, not to re-build established interfaces,” and it emphasizes that the practical problem being addressed is the current KE-level free-text “methods of measurement” field. The manuscript further proposes an “Observation” field linked to KEs, defined as comprising study design and measured effects, with additional linked attributes such as stressor, test system, assay endpoint, documentation, and measurement.

 At the same time, the revised wording now describes Observation as a diversity of evidence for any method yielding data to support a KE, and Figure 5 uses “has evidence / is evidence for” language for the relationship between observation and the claim it supports. The manuscript also notes that preliminary integration of Observation has already been presented in previous KER-centric evidence modeling efforts.

 This is where I still found the text somewhat difficult to understand. The use of the word “evidence” in the KE-linked Observation context naturally evokes the established AOP understanding of weight of evidence, which is usually centered on KERs rather than on KEs. The manuscript itself notes earlier that the AOP framework focuses on evidence for the linkages between critical KEs, while Methods2AOP is presented as a way to better structure documentation of methods used to measure KEs. Because of this, I think some readers may be uncertain whether Observation is intended primarily as: (1) a KE-linked empirical observation/measurement object that helps structure how a KE is assessed in experiments, or (2) a broader evidence object that is also meant to feed into KER-level evidence assembly and weight-of-evidence considerations.

 For me, the additional context from prior EMOD work helps resolve part of this ambiguity. In the EMOD prototype, Assay structures methods-of-measurement information, Observation holds empirically obtained KE measurements, and Evidence is explicitly a KER-level object that interlinks upstream and downstream observations linked to the respective KEs of the KER. With that broader context, the Methods2AOP proposal becomes easier to understand as a KE-level structuring layer for methods, assay, study design, and observation information, which could later support more transparent evidence assembly at the KER level. However, in the manuscript itself, that bridge remains mostly implicit.

 I therefore think the manuscript would benefit from a short paragraph making this boundary and relation to KERs more explicit. My understanding after reading the revision is that Methods2AOP is not proposing to replace the existing KER evidence evaluation structure of the AOP-Wiki, but rather to strengthen the structured documentation of KE-linked methods and observations so that these can better support interpretation, comparability, and potentially downstream evidence assembly. If that is indeed the intent, stating it directly would help substantially. It would also help distinguish between the current KE-page role (“how a KE is measured”) and the KER-page role (“how evidence for the linkage is evaluated”), which remain conceptually distinct in the current AOP-Wiki.

 Related to this, I still found the mapping between the conceptual entities in Figure 5 and the operationalized field groups in Figures 3-4 / the supplement somewhat implicit. My current reading is that Observation is a composite entity rather than a simple standalone field, and that its parts are represented in the supplementary file through grouped fields related to study design (Study Documentation, Study Subject Type, Study Husbandry/Care), assay/test system (Assay Documentation, Assay Characteristic), measurement (Assay Measurement). However, this is something the reader still has to infer, and it is still not clear where “effect” is represented, and how these pieces relate to the current KE-level free-text “How it is measured or detected” (and if relevant KER-level evidence) field. The worked examples are useful, but they do not yet fully resolve this conceptual mapping.

Is the work clearly and accurately presented and does it cite the current literature?

Yes

If applicable, is the statistical analysis and its interpretation appropriate?

Not applicable

Are all the source data underlying the results available to ensure full reproducibility?

No source data required

Is the study design appropriate and is the work technically sound?

Yes

Are the conclusions drawn adequately supported by the results?

Yes

Are sufficient details of methods and analysis provided to allow replication by others?

Yes

Reviewer Expertise:

Mechanistic toxicology, computational toxicology, AOP development

I confirm that I have read this submission and believe that I have an appropriate level of expertise to confirm that it is of an acceptable scientific standard, however I have significant reservations, as outlined above.

# Reviewer report: Reviewer response for version 1

**DOI:** 10.5256/f1000research.190643.r440513

**Published:** 2026-01-06

**Authors:** Alexandra Schaffert

**Affiliations:** 1 Tampere University, Tampere, Finland

The manuscript proposes a structured, ontology-supported framework (Methods2AOP) to improve how test method information is captured and linked to KEs in the AOP-Wiki, moving beyond the current free-text “How it is measured or detected” field. The approach is timely and well aligned with needs for interoperability, comparability, and reuse of method metadata across different evidence streams. A major potential strength of the proposed framework is its capacity to act as a machine-readable interface between toxicological data (e.g. NAM, high-throughput, regulatory, or academic datasets) and existing AOP content. By linking structured, ontology-backed method descriptors directly to KEs, the framework could enable more systematic identification of which experimental results inform which KEs and AOPs, thereby supporting AOP-guided interpretation, evidence assembly, and comparison across studies.

 The conceptual model and stakeholder-informed prioritization are a strong foundation, the main opportunities for strengthening the manuscript relate to clarifying intended scope and practical feasibility.

**Comments:**

 1. The manuscript introduces an “Observation” entity comprising study design and measured effects and describes this as supporting evidence for KEs. As written, it is not fully clear whether Observation is intended primarily as a bounded metadata structure to contextualize a method-KE linkage, or whether it is intended to capture potentially large volumes of study-level evidence instances over time. Clarifying this intent would help readers understand the anticipated scale, curation burden, and the role of external links versus internal storage. If evidence instances are in scope, it may be helpful to briefly acknowledge how volume and redundancy could be managed conceptually. Additionally, the framework uses “evidence for” language that may be interpreted as primarily confirmatory. In practice, capturing contradictory or null findings is important to avoid systematic positivity bias and to support weight-of-evidence thinking. The authors may wish to clarify whether contradictory/inconclusive observations are intended to be captured and, if so, indicate at a conceptual level how this would be represented.

 2. While the manuscript distinguishes “method” from “assay” and provides a functional description of “observation,” the practical boundaries between “method,” “assay,” “observation,” “study design,” and “effect” remain somewhat difficult to interpret as a reader, particularly in terms of what would constitute a single record of each type and how they relate in typical use. A short set of definitions (or a small glossary/box) clarifying what each term is intended to represent in this framework would be very useful. In addition, consider including concrete examples that demonstrate how these entities are put into practice, e.g., for a single KE, showing a typical current free-text “How it is measured or detected” description, how this information (and any additional relevant context) would map onto the proposed structured fields, and how the different entities (method, assay, observation, study design, effect) relate to each other within that example.

 3. The use of ontologies is a major strength for interoperability, however, ontologies evolve (terms can be updated, obsoleted, or replaced). It would strengthen the manuscript to briefly note this issue and potential traceability over time.

 4. The manuscript notes that case studies will be important for evaluating and refining the proposed framework. In this context, it may be helpful to clarify that the main value of such exercises lies not only in illustrating applicability, but in revealing practical issues that emerge when the framework is used by different contributors and across different examples. Experience with similar community-driven, ontology-based frameworks suggests that aspects such as field interpretation, level of detail, ontology term selection, and internal consistency are often only fully understood through repeated, real-world use rather than through a single demonstrative case. Briefly acknowledging that early application of the framework is likely to be an iterative learning process, informing clarification of guidance and incremental refinement of the structure over time, could help set appropriate expectations for future development.

Is the work clearly and accurately presented and does it cite the current literature?

Yes

If applicable, is the statistical analysis and its interpretation appropriate?

Not applicable

Are all the source data underlying the results available to ensure full reproducibility?

No source data required

Is the study design appropriate and is the work technically sound?

Yes

Are the conclusions drawn adequately supported by the results?

Yes

Are sufficient details of methods and analysis provided to allow replication by others?

Yes

Reviewer Expertise:

Mechanistic toxicology, computational toxicology, AOP development

I confirm that I have read this submission and believe that I have an appropriate level of expertise to confirm that it is of an acceptable scientific standard, however I have significant reservations, as outlined above.

# Response

**Published:** 2026-01-28

**Authors:** Clemens Wittwehr

**Affiliations:** Systems Toxicology, European Commission Joint Research Centre Ispra, Ispra, Italy

**General Response to Reviewer 2:**

 We thank the reviewer for their thorough, thoughtful, and constructive review. We particularly appreciate the reviewer’s recognition of the timeliness and potential value of the proposed Methods2AOP framework, especially its alignment with current needs for interoperability, comparability, and machine-readable linkage between test methods, evidence streams, and AOP content. The reviewer’s detailed comments and suggestions regarding scope clarification, conceptual boundaries, ontology use, and practical implementation have been very helpful and have guided us in strengthening the manuscript accordingly.

**Main comments**

**1. Clarification of the intended scope and role of the “Observation” entity**

 Reviewer 2 notes that it is not fully clear whether the proposed **Observation** entity is intended to be:

- a **bounded metadata structure** that contextualises a method–KE linkage, or
- a mechanism to capture potentially **large numbers of study-level evidence instances over time**.

They highlight that this distinction has implications for:

- anticipated scale,
- curation burden,
- and the balance between internal representation versus external linking.

They also raise an important conceptual point about **evidence polarity**. The current wording may be read as focusing on confirmatory evidence, whereas capturing **contradictory, null, or inconclusive findings** is essential to avoid positivity bias and to support weight-of-evidence thinking. They ask for clarification on whether such observations are in scope and, if so, how they would be represented at a conceptual level.

**Comment 1 Response:**

 We thank the reviewer for highlighting the need to be clear regarding the intention for the “Observation” entity. This is a central concept and we have made efforts to refine our description. In brief, the observation is the evidence supporting key event as derived from a test method. As the reviewer notes, this could be a large number of evidence and for any test method yielding data to support a KE, ample test method and assay data should be provided.

 Additions to manuscript shown in *italics *below:

- Section 3, Paragraph 2: “The proposed conceptual model, includes an “Observation” field defined as comprising information on study design and the measured effects (for which detailed inputs are summarized by the tiered data and detailed fields in Figures 3 & 4). Preliminary integration of “Observation” is already under development in the AOP-KB and has been presented as part of previous KER-centric Evidence Modeling prototyping efforts (Hench et al., 2024a; O'Brien and Yauk, 2022). *Challenges in integrating this field lie in the complexity of its parts (e.g., study design and effect which in turn also have their own ontological components) as “Observation” would include a diversity of evidence for any method that yields data to support a KE. By defining ontological terms appropriate per field, the Methods2AOP collaboration seeks to bound the diversity of metadata and simplify curation burden.*”

**2. Need for clearer boundaries and definitions between core entities**

 While acknowledging that the manuscript distinguishes between “method,” “assay,” and “observation,” Reviewer 2 finds that the **practical boundaries** between: method, assay, observation, study design, and effect remain difficult to interpret for a reader. In particular, it is not always clear:

- what constitutes a single record of each entity,
- how these entities relate to one another in typical use.

They suggest that this could be addressed through:

- a short set of definitions or a small glossary or boxed text, and
- a concrete example showing, for a single KE, how a current free-text “How it is measured or detected” description would map onto the proposed structured entities and fields, and how those entities interrelate

**Comment 2 Response:**

 Thank you for highlighting the need for a more concrete illustration of how each entity would be documented. We have revised the supplementary file where the granular definitions were provided to include some examples of documentation. Please refer to the newly updated supplementary file (Karmaus et al., 2025).

**3. Ontology evolution and long-term traceability**

 Reviewer 2 strongly supports the use of ontologies for interoperability, but notes that ontologies are **not static**. Terms may be updated, deprecated, or replaced over time. They suggest that briefly acknowledging this reality and the need for **traceability over time** would strengthen the manuscript and demonstrate awareness of long-term maintenance considerations

**Comment 3 Response:**

 This is a great point that we didn’t directly address but are in complete agreement. We have added a sentence to the *future needs* section to make this more explicit.

 Additions to manuscript summarized in *italics* below:

- Section 6, Paragraph 2: “Although information fields for methods have been identified and suggested here, the Methods2AOP collaboration members recognize the need to develop more case studies to evaluate and likely refine required information to ensure an easy and user-friendly approach and establish applicability to various contexts of use. *Furthermore, with the evergreen continuous evolution of ontologies it will be important to maintain versioning to support traceability over time in case of updated or deprecated terms.*”

**4. Case studies and iterative refinement of the framework**

 Reviewer 2 agrees that case studies will be important, but suggests going one step further in how they are framed. Beyond illustration, their main value lies in:

- revealing ambiguities in field interpretation,
- identifying inconsistencies in ontology term selection,
- exposing differences in contributor practices.

Drawing on experience from other community-driven, ontology-based frameworks, they note that many practical issues only emerge through **repeated real-world use**, not from a single demonstrative example. They therefore suggest explicitly acknowledging that early application of Methods2AOP will be an **iterative learning process**, informing refinement of guidance and incremental evolution of the framework over time.

**Comment 4 Response:**

 This is another point we absolutely agree with but didn’t explicitly state in the manuscript. We have made an addition in the manuscript, in *italics *below:

- Section 6, Paragraph 1: “ *Easing accessibility (e.g., via the simple tiered data input proposal) and committing to education for both input and retrieval of methods information, including revision of the AOPs Developer’s handbook, will be critical to the successful implementation, impact, sustainability, and growth of the AOP community. AOP authors and methods developers will need training to fill out the right information as the success of developing a new infrastructure will be an iterative learning process. Only by engaging and amassing repeated real-world use will we be able to identify ambiguities in field interpretation, inconsistencies in ontology term selection or expose differences in contributor practices. Lastly, and the benefit of embracing this new documentation will need to be well communicated to ensure participation and engagement*.”

# Reviewer report: Reviewer response for version 1

**DOI:** 10.5256/f1000research.190643.r440512

**Published:** 2025-12-31

**Authors:** Jinhee Choi, Elizabeth Dufourcq Sekatcheff

**Affiliations:** 1 University of Seoul, Seoul, South Korea; 2 Department of Risk Assessment, Agence Nationale de Sécurité Sanitaire de l'Alimentation, de l'Environnement et du Travail, Maisons-Alfort, France

A) 

 This manuscript, based on the activities of the Methods2AOP collaboration, proposes an approach to integrate and manage information on methods used to measure or detect Key Events in the AOP-Wiki by moving beyond the current free-text–driven format toward standardized metadata and a structured scheme supported by ontologies or controlled vocabularies. Overall, the attempt is timely and meaningful in that it aims to improve the applicability of NAMs and the reusability of data in AOP-based assessments; however, several core elements require further strengthening in terms of practical feasibility and regulatory and community use.

 Major comments

 1. The core of the manuscript is the proposal for structuring and standardizing method information, and readers will want to understand how this would function in practice within the AOP-KB. However, compared with the conceptual design and the presentation of candidate fields, the manuscript provides too few concrete application examples, which weakens the persuasiveness of the proposal. In particular, the manuscript would benefit from a more intuitive illustration of how the proposed metadata would be populated for a specific Key Event and how this would differ from, and improve upon, the current “How it is measured or detected” free-text field.

 2. Standardization of method information is already supported by multiple existing frameworks, including OECD test guidelines, guidance documents, and harmonised templates. Therefore, the manuscript should more clearly explain how this proposal relates to those existing standards and what additional problems it is intended to solve. While related systems are mentioned, further clarity is needed regarding why existing standards are insufficient and what role Methods2AOP is expected to play within the AOP-KB.

 3. Structured information systems often encounter challenges not at the design stage but during implementation and maintenance. Key issues include validation and quality control of entries, governance of terminology resources, and ensuring consistency across contributors, yet these aspects are not sufficiently addressed. Even if implementation is considered out of scope, the manuscript should provide at least a minimal operational and governance perspective, including a clearer view of which stakeholders might take on which roles in practice.

 4. The manuscript claims that the proposed structure can cover multiple study types and evidence streams, but the narrative and examples read as being weighted toward specific contexts such as in vitro. In addition, if regulatory relevance is a central motivation, the manuscript should more clearly articulate how structured method information could connect to concrete regulatory workflows, such as IATAs, Defined Approaches, weight-of-evidence evaluations, read-across justifications, and NGRA. Because the nature of required metadata can differ substantially across evidence streams, further justification is needed to support both the breadth and regulatory applicability of the proposal.

 Minor comments

 1. There are instances where the collaboration name and related abbreviations are not fully consistent throughout the manuscript. For example, in Section 2.2 the text uses “Methods2AOP” but also contains a sentence using “Method2AOP,” and the Note under Table 1 uses a spaced form such as “Methods 2AOP.”

 2. Several apparent spelling errors are present in the text. For example, “interpet,” “presentating,” “revison,” and “structrued” should be corrected.

 B)

 In this paper, the authors address a crucial topic on AOPs:  how to increase confidence in AOPs for regulatory purposes by integrating test methods in a standardized format in the AOP framework. The authors present here the result of a collaborative workshop and other initatives (EHLC, FAIR AOP cluster, ELIXIR), where they suggest an ontology based approach to adequately define method information needed.

 I have a few comments/questions on the choice of identified criteria that define test methods and on the practical implementation/developer guidance

 1.  In section 2.2, I was wondering whether it would be feasible to define information fields based on existing framework that establish test methods validation criteria (e.g., OECD GD 34) ? it would help to raise confidence in non standardized test methods in the AOP framework (e.g. literature) rather than having a separate section for non validated test methods, which I am concerned regulators will tend to overlook. 

 2. in section 2.3, it was not clear to me what to categorize as 'study type' as for eg, ecological study type can be performed with in vivo/in silico methods. As they are not mutually exclusive, how do you expect developers to fill information in the right section ?

 3. Overall, how do you envision the implementation of test methods in the AOPwiki database when there is no consensus yet on the terminology of KEs ? (numerous terminologies exist for e.g., oxidative stress, the term 'umbrella key event' was put forward to regroup several key events falling under the same scope...) I think this should be a problem to tackle first and the AOPwiki database would need cleaning to avoid duplication of KEs, accurate definitions of KE and associated test methods to enable correct linkage between AOPs and generation of AOP networks.

 4. Overall, do you plan to engage with european regulatory agencies (e.g., EFSA, ECHA) or member states authorities in this project ? I think it would be a great opportunity to ensure all regulatory needs are taken into account

 Overall, this is a great project and I'm looking forward to see the outcomes.

Is the work clearly and accurately presented and does it cite the current literature?

Yes

If applicable, is the statistical analysis and its interpretation appropriate?

Not applicable

Are all the source data underlying the results available to ensure full reproducibility?

No source data required

Is the study design appropriate and is the work technically sound?

Yes

Are the conclusions drawn adequately supported by the results?

Yes

Are sufficient details of methods and analysis provided to allow replication by others?

Yes

Reviewer Expertise:

Environmental toxicology

We confirm that we have read this submission and believe that we have an appropriate level of expertise to confirm that it is of an acceptable scientific standard, however we have significant reservations, as outlined above.

# Response

**Published:** 2026-01-28

**Authors:** Clemens Wittwehr

**Affiliations:** Systems Toxicology, European Commission Joint Research Centre Ispra, Ispra, Italy

**General Response to Reviewer 1:**

 We thank the reviewer for their thoughtful and constructive comments regarding the impact of Methods2AOP relative to existing standards, the operational and governance perspective, and the applicability of the proposal across evidence streams and regulatory contexts. The Methods2AOP collaboration seeks to bolster confidence in the use of AOPs by elaborating upon the documentation of methods used.

 With respect to existing standards, Methods2AOP does not seek to replace or duplicate established assay description frameworks or harmonized reporting templates developed in related initiatives. On the contrary, the collaboration explicitly considered these efforts and actively engaged with communities involved in assay documentation and standardization to ensure our proposal was complementary and interoperable. However, the current initiative described in our manuscript was focused on supporting knowledge integration across AOPs to allow for AOP-associated methods to be comparable, searchable, and reuseable within the AOP-Wiki and broader AOP-KB. Methods2AOP thus identified which method-related information should be captured in a structured, ontology-aware way to support AOP development and reuse, while remaining fully compatible with existing standards.

 From an operational and governance perspective, Methods2AOP deliberately avoids proposing new review, validation, or terminology governance structures. Instead, it is designed to piggyback on the established AOP-Wiki and OECD governance mechanisms. In practice, method-related information would be subject to the same contributor access rules, expert review processes, and quality expectations that already apply to AOP content. As AOPs progress toward higher levels of maturity and, ultimately, OECD endorsement, the stringency of review and quality control naturally increases, and any Methods2AOP-inspired structured method information would be reviewed accordingly. In this way, governance and quality assurance are inherited from existing processes rather than redefined.

 Regarding applicability across evidence streams and regulatory relevance, Methods2AOP is intentionally a bottom-up framework. It defines structured metadata elements that can capture method information across diverse study types (in vitro, in vivo, in silico, human, ecological), recognizing that the nature and granularity of relevant metadata will differ between these contexts. The framework does not attempt to define regulatory workflows such as IATAs, Defined Approaches, weight-of-evidence evaluations, read-across, or NGRA. Instead, it provides the structured building blocks that such workflows already rely on, enabling method information to be more transparent, comparable, and reusable when integrated into higher-level regulatory assessments. This is fully aligned with the broader AOP philosophy, in which modular, mechanistically grounded knowledge supports but does not prescribe regulatory decision-making.

 Overall, Methods2AOP should be understood as a pragmatic, enabling contribution that strengthens the interface between methods and AOPs, while remaining firmly grounded in existing standards, governance structures, and the established scope of the AOP framework and ultimately the regulatory use of AOP-based data.

**Main comments**

**1. Need for more concrete, worked examples**

 The reviewers emphasize that the core contribution of the manuscript is the proposal to move from free-text method descriptions toward structured, standardized metadata. While the conceptual design, candidate fields, and figures are clearly presented, the reviewers **find the proposal somewhat abstract** and would like to better understand how it would function in practice within the AOP-KB. In particular, they are looking for a more **intuitive illustration of how the proposed metadata would actually be populated** for a real Key Event, and how this would concretely improve on the current “How it is measured or detected” free-text field.

**Comment 1 Response:**

 We thank the reviewers for highlighting the need for a more concrete illustration of how the proposed structured metadata could be populated in practice. We agree that such examples demonstrate feasibility and provide added value. We have expanded the supplementary material (Supplementary Table 1) providing multiple worked examples within a single, versioned Excel file hosted on Zenodo. Each worked example is provided on a separate tab, allowing different methods and evidence streams to be illustrated while using the same underlying Methods2AOP metadata structure. Each tab populates only a subset of relevant metadata fields, intentionally demonstrating the principles of progressive disclosure and burden minimization. The examples show where ontology-backed annotation can be applied and where free-text entries and expert judgement remain appropriate, reflecting the current state of ontology coverage in toxicology. Importantly, these worked examples are illustrative rather than prescriptive. They are not intended to define mandatory fields, data-entry workflows, or user-interface design for the AOP-Wiki, but rather to demonstrate the types of structured information in practice. Specifically, the supplementary dataset now includes:

- two illustrative, non-exhaustive *in vitro* examples based on the human Cell Line Activation Test (hCLAT) and Epidermal Sensitization Assay (EpiSensA)
- an illustrative, non-exhaustive *in vivo* example based on electrocardiograph in rats
- an illustrative, non-exhaustive *in chemico* example based on Amino acid Derivative Reactivity Assay (ADRA)

Additions to manuscript summarized in italics below:

- Section 2.3 (after first paragraph): “…each of these study types would have their own groups of methods information needs. *In addition to listing the proposed metadata fields and ontology references, four illustrative, non-exhaustive worked examples demonstrating how selected fields can be populated for in vitro, in vivo, and in chemico test methods have been provided (Karmaus et al. 2025 [this is the updated reference for the supplementary file]). The worked examples intentionally do not populate all available fields, reflecting the principles of minimizing reporting burden while increasing transparency and easily interpretable information entry. Resources containing annotated methods using similar metadata fields that can support such data integration can include CAMERA (Collection of Alternative Methods for Regulatory Application; https://camera.niehs.nih.gov/) and CEBS (Chemical Effects in Biological Systems; https://cebs.niehs.nih.gov/cebs/).*”

**2. Clearer positioning relative to existing standards and frameworks**

 The reviewers note that method standardization is already supported by **several existing frameworks**, including OECD Test Guidelines, guidance documents, and harmonized templates. They therefore ask for a clearer explanation of how the Methods2AOP proposal **relates to these efforts**, why they are not sufficient on their own in the AOP-Wiki context, and what specific additional value Methods2AOP brings. While related initiatives are mentioned in the manuscript, the reviewer feels the relationship between these efforts and Methods2AOP should be made more explicit.

**Comment 2 Response:**

 The reviewers correctly note that multiple established frameworks already support assay description and standardization. Methods2AOP does not introduce a new or parallel standard but rather a proposed implementation of standardized vocabularies that is interoperable and complementary to existing frameworks. The added value of Methods2AOP lies in supporting comparability, searchability, and reuse of method information across AOPs, which is not the primary objective of existing assay-focused standards. The Methods2AOP suggested way of treating method information can serve as input for parties developing the AOP-Wiki 3.0 in the future, where relevant functionality can certainly be implemented.

 Additions to manuscript summarized in *italics *below:

- Abstract: “This paper reviews their work emphasizing the importance of linking detailed test method information and conceptually proposes how it may be included in the AOP knowledgebase *in alignment with existing assay documentation standards and governance frameworks.*”
- Section 3 (fourth paragraph): “Integrating more detailed methods information could strengthen confidence of the association between the method and the KE and consequently support the evidence for the regulatory endpoint. *In fact, the proposed ontologies support interoperability with existing frameworks and complement established documentation standards (e.g., OECD Harmonized Templates) which could increase the utility of AOPs.*”

**3. High-level operational and governance perspective**

 The reviewers highlight that structured information systems often encounter their main challenges **during implementation and long-term maintenance** rather than at the design stage. They specifically mention **validation and quality control of entries**, governance of terminology resources, and ensuring consistency across contributors. Even if implementation is considered out of scope, they ask for at least a minimal operational and governance perspective, including which stakeholders might realistically assume responsibility for these aspects.

**Comment 3 Response:**

 The reviewers raise important considerations regarding validation, quality control, and governance of structured information systems. Methods2AOP intentionally does not define new operational or governance structures. While this consideration is out of scope for the current work, the assumption for the collaboration during this effort was that any proposal would have to operate under the existing AOP-Wiki and OECD governance mechanisms, including contributor access control, expert review, and increasing levels of scrutiny as AOPs mature toward OECD endorsement. Structured method information inspired by Methods2AOP would therefore be subject to the same review expectations and quality assurance processes as other AOP content.

 Additions to manuscript summarized in *italics *below:

- Section 1.2: “The Methods2AOP collaboration suggest content and a conceptual documentation schema, presented herein for future consideration to integrate into the AOP-KB. *Accordingly, any structured method information inspired by Methods2AOP would be subject to the same governance, review, and quality control processes that already apply to AOP-Wiki content.*”

**4. Perceived in vitro bias and regulatory applicability**

 The reviewers observe that although the manuscript claims applicability across multiple study types and evidence streams, the narrative and examples appear weighted toward **in vitro** contexts. They ask for clearer justification that the proposed structure can cover diverse evidence streams (in vivo, in silico, human, ecological) and for a more explicit explanation of how structured method information would connect to concrete regulatory workflows such as IATAs, Defined Approaches, weight-of-evidence evaluations, read-across, and NGRA.

**Comment 4 Response:**

 The reviewers note a perceived emphasis on in vitro contexts and ask for clearer links to regulatory workflows. The use of in vitro examples primarily reflects their suitability for illustration rather than a limitation of the framework. Methods2AOP is designed to accommodate method information across study types, recognizing that metadata needs differ between in vitro, in vivo, in silico, human, and ecological contexts. We have provided more detailed examples of other study types to these ends in the updated Supplementary File (Karmaus et al. 2025). The in vitro study type remains as the illustrative example in the manuscript given the overwhelming abundance of in vitro assays used as evidence for mechanistic KEs.

 The Methods2AOP collaboration does not aim to define the impact of methods documentation within the AOP infrastructure on regulatory workflows such as IATAs, Defined Approaches, weight-of-evidence evaluations, read-across, or NGRA. Rather, it proposes structured and transparent method documentation which bolsters the use of AOPs in regulatory contexts (which would in turn include applying AOPs as lines of evidence in a multitude of contexts including the workflows listed. We have clarified this in the manuscript.

 Additions to manuscript summarized in *italics *below:

- End of Section 3: “ *Consistent with the broader AOP framework, the proposed structuring of method information is intended to bolster confidence in the application of AOPs which can in turn support higher-level regulatory applications such as Integrated Approaches to Testing and Assessment, Defined Approaches, and weight-of-evidence evaluations, without prescribing how such workflows should be constructed.*”
